# B-cell precursors (hematogones) in CD34+ hematopoietic stem cell collections: a single-center experience

**DOI:** 10.31744/einstein_journal/2022CE0018

**Published:** 2022-09-05

**Authors:** Daniel Mazza Matos

**Affiliations:** 1 Centro de Hematologia e Hemoterapia do Ceará Fortaleza CE Brazil Centro de Hematologia e Hemoterapia do Ceará, Fortaleza, CE, Brazil.

Dear Editor,

CD34+ hematopoietic stem cell (CD34+ HSC) enumeration plays a significant role in feasibility of hematopoietic stem cell transplantations (HSCT). Currently, most centers collect a minimum of 2.0 and 4.0 million viable CD34+ HSC per kilogram of recipient’s weight for autologous-HSCT and allogeneic-HSCT, respectively. While the effect of CD34+ HSC dose on the clinical outcome (mainly in allogeneic-HSCT) is still poorly defined, there is a consensus that receiving a suboptimal dose of CD34+ HSC is associated with delayed neutrophil and platelet recovery and, eventually, inferior survival related to late engraftment.

In this context, B-cell precursors, morphologically known as hematogones, are sometimes found in mobilized leukapheresis used for HSCT.^(1)^ Eventually, the number of B-cell precursors in bags programmed to be infused in patients submitted to HSCT is significant, and their presence raises the question whether a suboptimal number of CD34+ HSC could have been collected. Besides, it is not clear how the presence of B-cell precursors in leukapheresis collections should be better reported by the flow cytometry laboratory in events of marginal CD34+ cell content, nor whether the recipients of such data would necessarily know how to interpret and act upon it.

In this letter, we intended to achieve three goals. First, we reviewed our flow cytometry data regarding the enumeration of CD34+ HSC to obtain frequency and number of B-cell precursors in diverse materials. Second, we briefly discussed the relevance of B-cell precursors in the context of HSCT and what is the best strategy to identify them; we anticipate it has not been defined yet. Third, we gave some suggestions on how to report flow cytometry data in the presence of B-cell precursors, aiming to help the transplant physician.

Between January 2019 and August 2021, we received at the Cell Processing Center (CPC - HEMOCE) samples from 240 subjects for routine CD34+ HSC enumeration, namely: 270 mobilized peripheral blood, 265 leukapheresis and 14 umbilical cord blood.

For CD34+ HSC quantification, the single-platform BD^TM^ Stem Cell Enumeration Kit was prepared according to manufacturer’s recommendations, as described elsewhere.^(2)^ To detect B-cell precursors, we designed an extra-gate to include events characterized by CD34+, CD45+ of low expression and very low light forward scatter and side scatter, which comprise the typical immunophenotype of B-cell precursors. Although not strictly necessary,^(1)^ in some particular cases we added an anti-CD19 APC antibody to our standard single-platform BD^TM^ Stem Cell Enumeration Kit (anti-CD45 FITC, anti-CD34 PE, 7-AAD) to improve the precision of B-cell precursor quantification (Figure 1).

**Figure 1:**
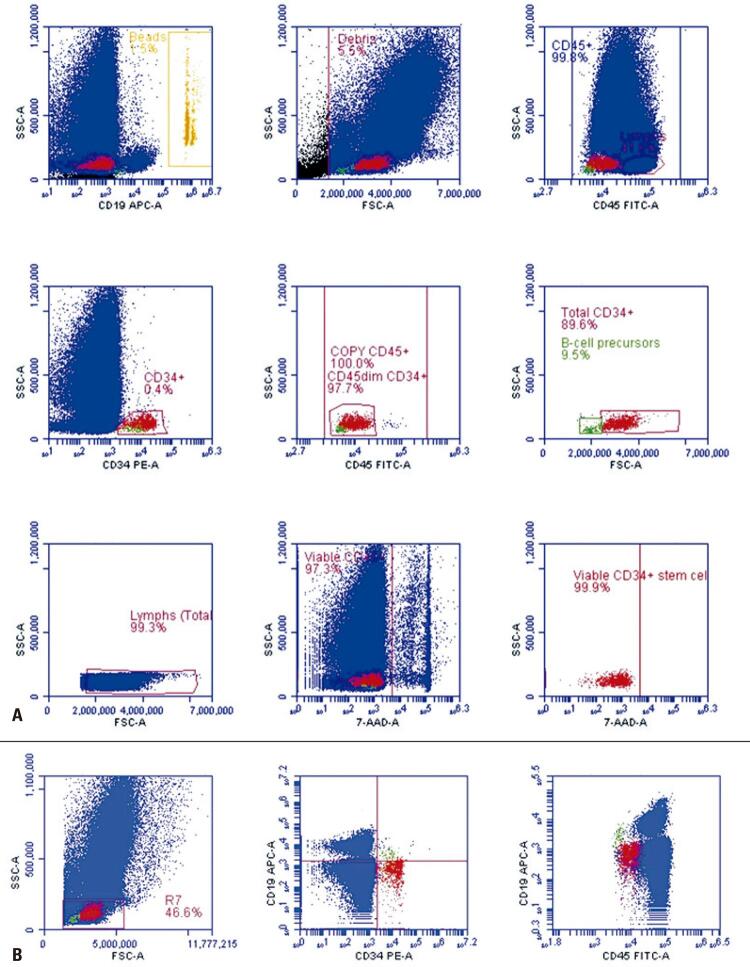
(A) Enumeration of viable CD34+ HPC and B-cell precursors with the use of single-platform International Society of Hematotherapy and Graft Engineering - ISHAGE-based protocol. Data were collected on a BD FACSVia™. A fresh apheresis sample was stained with the BDTM Stem Cell Enumeration Kit containing anti-CD45 FITC, anti-CD34 PE and 7-AAD. We added anti-CD19 APC during the staining procedure. Viable (7-AAD negative) CD34+ HPC were identified by means of Boolean gating (plots 1 to 9, from left to right). In plot 6 (second line, right plot) we designed an extra-gate (green) to include B-cell precursors. Notice the very low forward scatter and side scatter of B-cell precursors (green) when compared with CD34+ HPC (red). Presence of 9.5% of B-cell precursors; (B) Plot 1 (first line, left): ‘lymphocyte gate’ (R7). In plot 2 (first line, middle) notice the presence of four cell populations: (1) LL (CD19‒/CD34‒): T-lymphocytes and NK- lymphocytes (blue); (2) UL (CD19+/CD34‒): B-lymphocytes (blue); UR (CD19+/CD34+): B-cell precursors (green); LR (CD19‒/CD34+): CD34+ HPC (red). In plot 3 (first line, right) notice again the presence of the four cell populations: B-cell precursors (green) exhibited positivity for CD19. The expression of CD45 in B-cell precursors (green) is low when compared to CD34+ HPC (red). The strategy to identify B-cell precursors was the following: initially, an extra-gate was designed on plot 6, figure 1A to include CD34+/very low forward scatter/very low side scatter cells. Afterwards, we “painted” the CD34+/very low forward scatter/very low side scatter cells with a green color. Next, the four populations of plot 2, figure 1B (the quadrant plot), which are fractions of viable lymphocyte gate (R7, plot 1, Figure 1B), could be identified, as previously described. The purpose of the quadrant plot was only to verify if the population of cells characterized by CD34+/very low forward scatter/very low side scatter population (included in the extra-gate of plot 6, Figure 1A) were really CD19+ cells; in this case, B-cell precursors

SSC: side scatter; FSC: forward scatter.

The frequency and the mean number of B-cell precursors are shown in table 1. We found B-cell precursors in 38 out of 549 (6.9%) samples, namely: 3 out of 14 (21.4%) umbilical cord blood, 31 out of 265 (11.7%) leukapheresis, and 4 out of 270 (1.5%) of mobilized peripheral blood.

**Table 1:** Frequency, mean and range of B-cell precursors in varied materials

Materials	Total number	Samples with B-cell precursors	Frequency of B-cell precursors (%)	Mean and range of B-cell precursors (%)
Mobilized peripheral blood	270	4	1.5	6.9 (5.3-8.0)
Leukapheresis	265	31	11.7	8.5 (1.0-44.1)
Umbilical cord blood	14	3	21.4	7.1 (3.7-12.9)
Total	549	38	6.9	7.5 (1.0- 44.1)

Of note, we had access to diagnosis of 229 subjects. The distribution of B-cell precursors based on the primary diagnosis was: plasm cell myeloma = 15 out of 126 subjects (12%); Hodgkin lymphoma = 7 out of 44 subjects (16%); diffuse large B-cell lymphoma, not otherwise specified (NOS) = 1 out of 10 subjects (10%); chronic lymphocytic leukemia = 1 out of 1 subject (100%); acute myeloid leukemia, NOS = 1 out of 1 subject (100%).

We found no B-cell precursors in 14 subjects with non-Hodgkin’s lymphoma, NOS; 10 subjects with mantle cell lymphoma; 5 with germ cell tumors; 3 with follicular lymphoma; 3 with acute promyelocytic leukemia with *PML-RARA*; 1 with peripheral T-cell lymphoma, NOS; 1 with anaplastic large cell lymphoma, ALK-positive; 1 with anaplastic large cell lymphoma, ALK-negative; 1 with angioimmunoblastic T-cell lymphoma; 1 with plasmablastic lymphoma; 1 with T-cell/histiocyte-rich large B-cell lymphoma; 1 with B-lymphoblastic leukemia/lymphoma, NOS; 1 with B-lymphoblastic leukemia/lymphoma, BCR-ABL 1; 1 with ovarian germ cell tumor; 1 with POEMS syndrome; 1 with extraosseous plasmacytoma and 1 with amyloidosis.

Ondrejka et al.^(3)^ analyzed 274 leukapheresis samples and found 61% harboring B-cell precursors. Patients (n=147) received stem cell infusions that were negative, mixed, or positive for B-cell precursors (of note: negative = <0.01%, positive = ≥0.01%, mixed = combination of collection products, some positive and some negative for B-cell precursors). Through the multivariate analysis for the variable ‘Predictors for Neutrophil Engraftment’, the authors asserted that patients in the mixed and positive groups had a marginally greater likelihood of neutrophil engraftment than those with negative hematogones. The authors also stated hematogones had no impact on platelet engraftment.

Notwithstanding, we believed these conclusions are hasty and cannot be taken as definitive. It seems the main problem here is that we indeed do not have any available data that allow a sub-analysis of the positive group regarding the number of CD34+ HSC per kilogram of recipient’s weight, not considering the presence of B-cell precursors. What one really wants to know is whether patients that received bags with a suboptimal number of CD34+ HSC (due to the presence of B-cell precursors) had any impact in the time of engraftment. Unfortunately, as expected, the data presented by the study do not provide this information.

Therefore, it is necessary to turn to basic research and see whether we can obtain some insightful information from there.

Immune reconstitution after allogeneic-HSCT has been extensively studied with a focus on T-cell reconstitution. Only limited information is available about B-cell reconstitution. Imamura et al.^(4)^ have previously showed that pro-B cells (CD34+ CD38+ CD10+ CD19+ CD13– Lin–), sorted from human bone marrow or G-CSF-mobilized peripheral blood, exhibited only B-lymphoid reconstitution in irradiated NOD/SCID/β2^–/–^ mice, while human CD34+ Lin^–^ cells have a broad capacity to differentiate into myeloid cells, B cells, and NK cells. Moreover, these pro-B cells did not exhibit self-renewal or long-term culture initiating-cell capacity, indicating the mobilized cells had characteristics of B lineage-committed progenitors.^(4)^

These data appear to be indirectly confirmed by studies where combined bone marrow and peripheral blood progenitor cell autografts are used for HSCT.^(5)^ In fact, harvesting bone marrow graft to provide an adequate number of CD34+ cells for transplant (when such number has not been reached with the initial peripheral blood progenitor cells collection) indicates the larger the proportion of contribution by bone marrow to combined grafts (bone marrow + peripheral blood progenitor cells), the longer the period till neutrophil engraftment.^(5)^ This is probably not fortuitous, given the fact that the proportion of B-cell precursors is typically higher in bone marrow collections when compared with peripheral blood progenitor cell collections. On the other hand, in peripheral blood progenitor cell collections, the proportion of multipotent progenitors is higher than in bone marrow collections.^(6)^ Hence, it is not far-fetched to state the high proportion of B-cell precursors in bone marrow could explain the limited capacity of immune reconstitution of that source, which could also justify the longer time to neutrophil engraftment in studies of combined (bone marrow + peripheral blood progenitor cells) cell autografts.^(5,7)^

Taken together, the data are currently insufficient and do not allow for definitive conclusions to be drawn about the role of B-cell precursors in immune reconstitution after HSCT, although there are some hints that B-cell precursors are involved only in B-cell reconstitution; thus, they should be excluded from CD34+ HSC counts.

## So, where do we stand?

At our center we objectively draw attention to the presence of B-cell precursors in flow cytometry reports whenever the total number of CD34+ HSC, excluding the presence of B-cell precursors, is less than 2.0 x 10^6^ cells per kilogram of recipient’s weight. Therefore, to alert the transplant physician about the possibility that - due to the presence of B-cell precursors - the bag destined for the HSCT may contain a suboptimal number of CD34+ HSC, we suggest the laboratories three simple actions as general recommendation.

First, for the quantification of B-cell precursors, the addition of an anti-CD19 antibody (Figure 1) in the standard tube (anti-CD45, anti-CD34, 7-AAD) to increase the precision of quantification, at least for some cases where the delimitation of small B-cell precursor populations, based only on physical properties (forward scatter and side scatter) and low intensity CD45 expression, can be difficult and less accurate. Second, the percentage of B-cell precursors and the number of CD34+ HPC non-B-cell precursor cells per kilogram of recipient’s weight in the bag should be reported. Third, a detailed report, preferably including some brief considerations about what the presence of B-cell precursors means, could help the transplant physician make better clinical decisions. In our center, we systematically include in our flow cytometry report, the following advice, as a final note:

“Presence, among the total number of CD34+ cells in the sample, of *x*% of cells characterized by very low cell size (forward scatter) and very low internal complexity (side scatter), associated with positivity for CD45 of low intensity, probably corresponding to the population of B-cell precursors (hematogones). Note that, with the exclusion of the aforementioned population of B-cell precursors, the corrected value for viable CD34+ cells is y x 10^6^cells/kg. Of note, the literature has no conclusive clinical evidence on what to do in the presence of large numbers of B-cell precursors in the bags for transplants. Thus, it is reasonable, given the little information available, to not consider B-cell precursors as equivalent to CD34+ HSC, and for transplant purposes, to take the number expressed by the corrected value for viable CD34+ cells”.

Finally, we would like to make some comments regarding standardization and validation. Today we do not know if the inclusion of CD19 antibody (CD19-based gating) (Figure 1) is better than the gating of B-cell precursors based only on the physical characteristics (forward scatter/side scatter-based gating) of these cells, as originally described by Thiago et al.^(1)^ If CD19-based gating proved to be better, other questions related to the standardization also need to be answered, such as what is the best CD19 antibody-fluorochrome combination?; what is the best CD19 clone to be selected?.

We believe these issues should be addressed in future studies, and once standardization of the method has been defined, the validation process can be started, probably in a landscape of multicenter cooperation.
